# Distúrbios de Condução após o Implante Transcateter de Válvula Aórtica: Desafio para mais 20 Anos?

**DOI:** 10.36660/abc.20220619

**Published:** 2022-10-05

**Authors:** Antonio Hélio Pozetti, Henrique Barbosa Ribeiro

**Affiliations:** 1 Hospital das Clínicas Faculdade de Medicina Universidade de São Paulo São Paulo SP Brasil Instituto do Coração do Hospital das Clínicas da Faculdade de Medicina da Universidade de São Paulo , São Paulo , SP – Brasil; 2 Hospital Samaritano Paulista São Paulo SP Brasil Hospital Samaritano Paulista , São Paulo , SP – Brasil

**Keywords:** TAVI, Marca-passo, Distúrbios de Condução

O implante transcateter de válvula aórtica (TAVI) é um procedimento bem estabelecido para o tratamento da estenose aórtica grave, independente do risco cirúrgico para pacientes idosos. ^1^ Desde sua introdução há duas décadas houve grandes avanços tecnológicos nos dispositivos, que aliados a novas técnicas de implante trouxeram reduções significativas nas taxas de complicações periprocedimento, impulsionando sua maior adoção mundialmente. No entanto, a incidência dos distúrbios de condução apresentou redução modesta, permanecendo como a complicação mais frequente após o TAVI, ^2 - 4^ o que contribui para o aumento da permanência hospitalar, dos custos e da piora dos desfechos clínicos a curto e longo prazos. ^4 , 5^ Além disso, a abordagem dos distúrbios de condução ainda tem grande variação entre os centros, principalmente em relação ao manejo do bloqueio de ramo esquerdo (BRE) novo, bloqueio atrioventricular avançado (BAV) pós procedimento e bloqueio de ramo direito (BRD) prévio, traduzido em taxas variáveis de implante de marca-passo definitivo (MP). ^3^ Entre os pacientes que receberam MP após o TAVI também há grande variabilidade com respeito à sua dependência (estimulação ventricular) no seguimento.

Nesta edição do jornal, Pinto et al. ^6^ avaliaram a incidência de distúrbios de condução, preditores e a taxa de dependência do MP, em uma população de 340 pacientes consecutivos submetidos a TAVI. ^6^ Os distúrbios de condução ocorreram em mais de 50% dos pacientes pós procedimento, sendo o BRE o mais frequente (32%), com resolução espontânea em 56% deles após 6 meses. A taxa global de implante de MP foi de 18,5%, sendo o BRD prévio seu principal preditor. Entre os pacientes que necessitaram de MP, as principais razões foram BAV avançado (60,3%), seguido do BRE com BAV de baixo grau (22%). De maneira interessante, houve grande variação na porcentagem de estimulação ventricular entre os pacientes que receberam MP, sendo de 83% nos pacientes com BAV avançado (BAVT e Mobitz II) e de apenas 2% naqueles implantados por BRE e BAV de baixo grau (BAV de primeiro grau e Mobitz tipo I). Os resultados deste estudo reforçam os dados da literatura que demonstram a elevada incidência dos distúrbios de condução em cerca da metade dos pacientes após o TAVI e a necessidade de MP definitivo em quase 20% deles. ^2 - 4 , 7^ Contudo, alguns aspectos deste estudo merecem uma reflexão.

Em primeiro lugar, a avaliação pré-procedimento é fundamental para identificarmos fatores de risco para distúrbios de condução e auxiliar a estratégia do operador. Assim como demonstrado por Pinto et al., ^6^ a presença de BRD prévio (~10% dos pacientes) ^8^ é o principal fator de risco para implante de MP pós TAVI, aumentando sua incidência em 3 a 4 vezes, ^8 , 9^ sendo também um preditor de mortalidade pós-procedimento. ^9^ No presente estudo o BRE e o BAV de primeiro grau prévios não se correlacionaram com maior necessidade de MP, diferentemente de outros autores que em número maior de pacientes, demonstraram que o BRE pode ter impacto na necessidade de MP nos primeiros 30 dias, mas não no seguimento após 30 dias, porém sem impacto em mortalidade. ^8 , 10^

Um segundo ponto importante refere-se ao procedimento, no qual alguns aspectos modificáveis também podem influenciar nas taxas de distúrbios de condução. Por exemplo, metade dos distúrbios de condução ocorrem antes do implante da válvula, principalmente durante a pré-dilatação, sugerindo alguma correlação com valvoplastia por balão, ^11^ como também evidenciado no presente estudo. Além disso, foi demonstrado que as válvulas de nova geração e técnicas para implantes mais altos no anel propiciaram reduções significativas nas taxas de MP para <10%. ^7 , 8 , 12^ De fato, Pinto et al. ^6^ demostram uma redução de quase 50% com as próteses de nova geração, e na presença de bioprótese cirúrgica disfuncionante ( *valve-in-valve* ). ^13^

Ao final do procedimento um eletrocardiograma (ECG) de 12 derivações deve ser realizado para determinar a conduta frente aos eventuais distúrbios de condução, bem como a monitorização contínua por 12-24 horas. ^2^ Assim como visto por Pinto et al. ^6^ o principal distúrbio de condução é o BRE, sendo que 10-15% evoluem para MP no primeiro ano, ^9^ reforçando a importância da monitorização ambulatorial destes casos. Posto a isso, recomenda-se a avaliação seriada dos ECG, sendo que em casos de aumento dos intervalos PR ou QRS > 20 ms, especialmente na presença de PR>240 ms e QRS>150 ms pode-se indicar o implante de MP profilático pelo risco de morte súbita e BAV avançado. ^2^ No estudo de Pinto et al. ^6^ 22% das indicações de MP foram por BRE combinado com BAV primeiro grau, porém este grupo apresentou apenas 2% de estimulação ventricular em 1 ano, demonstrando a dificuldade em conduzir este tipo de paciente bem como a real necessidade do marcapasso em determinadas circunstâncias, uma vez que apesar da baixa estimulação, ela tenha ocorrido durante episódios paroxíticos de BAV avançado ou bradicardia extrema com risco de vida.

Apesar de duas décadas de avanço tecnológico e melhora dos resultados da TAVI, os distúrbios de condução ainda permanecem como principal complicação pós TAVI. Diversos estudos nos últimos anos contribuíram para a identificação dos fatores de risco, permitindo uma redução das taxas de MP e tem auxiliado no manejo desses pacientes. Embora o estudo de Pinto et al. ^6^ apresente algumas limitações (retrospectivo, observacional e unicêntrico), ele reforça o BRD prévio como principal fator de risco para necessidade de MP e traz uma reflexão sobre sua indicação para pacientes com BRE e BAV primeiro grau, onde estudos prospectivos como o PROMOTE ( *clinicaltrials.org. NCT: 04139616)* irão avaliar algoritmos específicos de manejo dos distúrbios de condução após o TAVI. Nesses pacientes, talvez a avaliação eletrofisiológica do sistema de condução, mesmo no período periprocedimento, poderá auxiliar no manejo e indicação mais precisa do MP. ^14^
