# Supplementary material for: Distúrbios de Condução após o Implante Transcateter de Válvula Aórtica: Desafio para mais 20 Anos?
Source: Arq Bras Cardiol. 2022 Oct 5;119(4):531–2. [Article in Portuguese] doi: 10.36660/abc.20220619 (PMC9563871; doi:10.36660/abc.20220619)
Supplement: Translation

# Translation: Conduction Disturbances Associated with Transcatheter Aortic Valve Implantation: Challenge for another 20 Years?

**Authors:** Antonio Hélio Pozetti, Henrique Barbosa Ribeiro

**Affiliations:** 1 Brazil Instituto do Coração do Hospital das Clínicas da Faculdade de Medicina da Universidade de São Paulo, São Paulo, SP – Brazil; 2 Brazil Hospital Samaritano Paulista, São Paulo, SP – Brazil

**Keywords:** TAVI, Pacemaker, Conduction Disturbances

Transcatheter aortic valve implantation (TAVI) is a well-established procedure for treating severe aortic stenosis in elderly patients, regardless of the surgical risk.^1^ Since its introduction two decades ago, there have been major technological advances in devices, which, combined with new implantation techniques, have brought significant reductions in periprocedural complication rates, leading to their greater adoption worldwide. However, the incidence of conduction disorders showed a modest reduction, remaining the most frequent complication after TAVI,^2 - 4^ which contributes to the increase in hospital stay, costs and the worsening of short and long-term clinical outcomes.^4^ In addition, the approach to conduction disorders still varies greatly between centers, especially regarding the management of new left bundle branch block (LBBB), post-procedure advanced atrioventricular block (AVB) and previous right bundle branch block (RBBB), translated into variable rates of permanent pacemaker (PM) implantation.^3^ Among patients who received PM after TAVI, there is also great variability regarding their dependence (ventricular pacing) at follow-up.

In this journal edition, Pinto et al.^6^ evaluated the incidence of conduction disorders, predictors and the rate of PM dependence in a population of 340 consecutive patients undergoing TAVI.^6^ Conduction disorders occurred in more than 50% of post-procedure patients, with LBBB being the most frequent (32%), showing spontaneous resolution in 56% of them after 6 months. The overall PM implant rate was 18.5%, with prior RBBB being its main predictor. Among the patients who required PM, the main reasons were advanced AVB (60.3%), followed by LBBB with low-degree AVB (22%). Interestingly, there was a wide variation in the percentage of ventricular pacing among patients who received PM, being 83% in patients with advanced AVB (Advanced AVB and Mobitz Type II) and only 2% in those implanted with LBBB and low-degree AVB (first-degree AVB and Mobitz type I).^2 - 4^ However, some aspects of this study deserve reflection.

First, pre-procedure assessment is essential to identify risk factors for conduction disorders and assist the operator’s strategy. As demonstrated by Pinto et al.^6^ the presence of previous RBBB (~10% of patients)^8^ is the main risk factor for PM implantation after TAVI, increasing its incidence by 3 to 4 times,^8^ it is also a predictor of post-procedure mortality.^9^ In the present study, previous LBBB and first-degree AVB were not correlated with a greater need for PM, unlike other authors who, in a larger number of patients, have shown that LBBB can have an impact on the need for PM in the first 30 days, but not in the follow-up after 30 days, despite no impact on mortality.^8^

A second important aspect is a procedure in which some modifiable aspects can also influence the rates of conduction disorders. For example, half of the conduction disturbances occur before valve implantation, mainly during predilation, suggesting some correlation with balloon valvuloplasty,^11^ as also evidenced in the present study. In addition, new generation valves and techniques for higher implantantion in the annulus have significantly reduced PM rates to <10%.^7 , 8 , 12^ In fact, Pinto et al.^6^ showed a reduction of almost 50% with new generation prostheses and in the presence of dysfunctional surgical bioprosthesis (valve-in-valve).^13^

At the end of the procedure, a 12-lead electrocardiogram (ECG) should be performed to determine the management of any conduction disturbances and continuous monitoring for 12-24 hours.^2^ As seen by Pinto et al.^6^ the main conduction disorder is LBBB, with 10-15% progressing to PM in the first year,^9^ reinforcing the importance of outpatient monitoring of these cases. Therefore, serial ECG evaluation is recommended, and in cases of increased PR or QRS intervals > 20 ms, especially in the presence of PR>240ms and QRS>150ms, prophylactic PM implantation may be indicated by the risk of sudden death and advanced AVB.^2^ In the study by Pinto et al.^6^ 22% of the PM indications were for LBBB combined with first-degree AVB. However, this group had only 2% of ventricular pacing at one year, demonstrating the difficulty in managing this type of patient as well as the real need for a PM in certain circumstances since, despite low stimulation, it has occurred during paroxysmal episodes of advanced AVB or extreme bradycardia that are life-threatening.

Despite two decades of technological advancement and improvement in TAVI results, conduction disturbances remain the most frequent complication. Several studies in recent years have contributed to identify risk factors, allowing a reduction in PM rates and has helped in the management of these patients. Although the study by Pinto et al.^6^ presents some limitations (retrospective, observational and unicentric), it reinforces previous RBBB as the main risk factor for the need of PM, and it brings a reflection on its indication for patients with LBBB and first-degree AVB, where prospective studies such as PROMOTE (clinicaltrials. org. *NCT: 04139616)* will evaluate specific algorithms for the management of conduction disorders after TAVI. In such patients, perhaps the electrophysiological assessment of the conduction system, even in the periprocedural period, may help in the management and the more precise indication of PM.^14^
